# Sitting less elicits metabolic responses similar to exercise and enhances insulin sensitivity in postmenopausal women

**DOI:** 10.1007/s00125-021-05558-5

**Published:** 2021-09-12

**Authors:** Carlijn M. E. Remie, Georges E. Janssens, Lena Bilet, Michel van Weeghel, Bernard M. F. M. Duvivier, Vera H. W. de Wit, Niels J. Connell, Johanna A. Jörgensen, Bauke V. Schomakers, Vera B. Schrauwen-Hinderling, Joris Hoeks, Matthijs K. C. Hesselink, Esther Phielix, Riekelt H. Houtkooper, Patrick Schrauwen

**Affiliations:** 1 Department of Nutrition and Movement Sciences, NUTRIM School of Nutrition and Translational Research in Metabolism, Maastricht University Medical Center, Maastricht, the Netherlands; 2 Laboratory Genetic Metabolic Diseases, Amsterdam Gastroenterology, Endocrinology, and Metabolism, Amsterdam Cardiovascular Sciences, Amsterdam UMC, University of Amsterdam, Amsterdam, the Netherlands; 3 Core Facility Metabolomics, Amsterdam UMC, University of Amsterdam, Amsterdam, the Netherlands; 4 Public Health and Primary Care, KU Leuven, Leuven, Belgium; 5 Department of Radiology and Nuclear Medicine, Maastricht University Medical Center, Maastricht, the Netherlands

**Keywords:** Clinical trial, Exercise, Insulin sensitivity, Metabolomics, Muscle metabolism, Sedentary time, Sitting, Sitting less

## Abstract

**Aims/hypothesis:**

In our current society sedentary behaviour predominates in most people and is associated with the risk of developing type 2 diabetes. It has been suggested that replacing sitting time by standing and walking could be beneficial for individuals with type 2 diabetes but the underlying mechanisms are unknown and direct comparisons with exercise are lacking. Our objective was to directly compare metabolic responses of either sitting less or exercising, relative to being sedentary.

**Methods:**

We performed a randomised, crossover intervention study in 12 overweight women who performed three well-controlled 4 day activity regimens: (1) sitting regimen (sitting 14 h/day); (2) exercise regimen (sitting 13 h/day, exercise 1 h/day); and (3) sitting less regimen (sitting 9 h/day, standing 4 h/day and walking 3 h/day). The primary outcome was insulin sensitivity measured by a two-step hyperinsulinaemic–euglycaemic clamp. We additionally performed metabolomics on muscle biopsies taken before the clamp to identify changes at the molecular level.

**Results:**

Replacing sitting time by standing and walking over 4 days resulted in improved peripheral insulin sensitivity, comparable with the improvement achieved by moderate-to-vigorous exercise. Specifically, we report a significant improvement in peripheral insulin sensitivity in the sitting less (~13%) and the exercise regimen (~20%), compared with the sitting regimen. Furthermore, sitting less shifted the underlying muscle metabolome towards that seen with moderate-to-vigorous exercise, compared with the sitting regimen.

**Conclusions/interpretations:**

Replacing sitting time by standing and walking is an attractive alternative to moderate-to-vigorous exercise for improving metabolic health.

**Trial registration:**

ClinicalTrials.gov NCT03912922.

**Graphical abstract:**

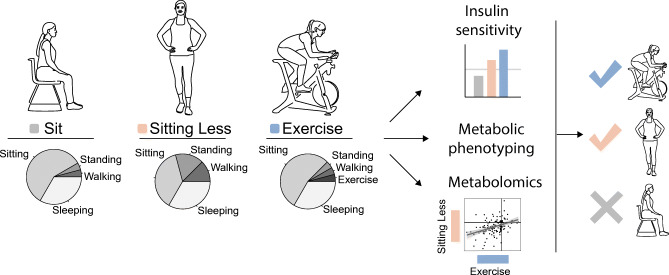

**Supplementary Information:**

The online version contains peer-reviewed but unedited supplementary material available at 10.1007/s00125-021-05558-5.

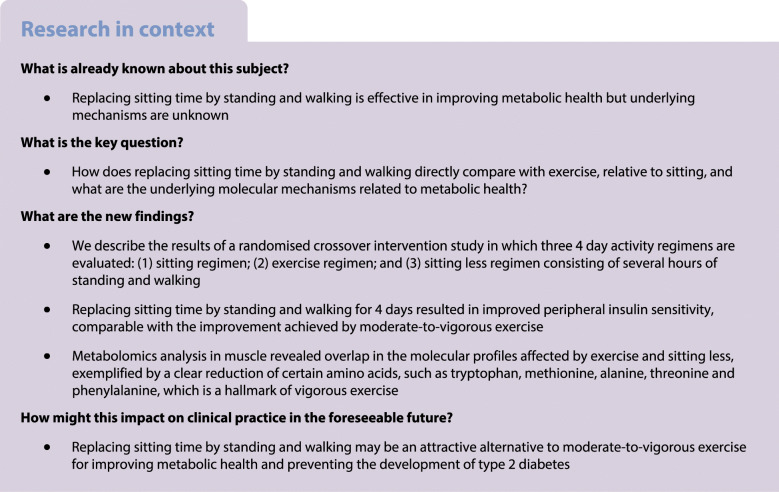



## Introduction

Exercise is the cornerstone of effective prevention and treatment of type 2 diabetes, yet the compliance for maintaining an active lifestyle with regular moderate-to-vigorous physical activity is low in the general European and US adult population [1, 2]. Compliance is even lower in people diagnosed with type 2 diabetes or adults at risk of developing this disease [3]. Conversely, sedentary behaviour is a risk factor for type 2 diabetes, the metabolic syndrome, CVD and all-cause mortality [4–6]. It has been recently suggested that reducing sedentary time, regardless of time spent in exercise activities, could be sufficient to improve glucose homeostasis in type 2 diabetes. Namely, human intervention studies in which sedentary time is replaced with standing [7, 8], walking [7–11] or short exercise bouts [11] have shown beneficial effects on postprandial plasma glucose and insulin levels. We contributed to this field by showing that improvements in glycaemic control are evident after a 4 day intervention in which two sitting replacement strategies (referred to as ‘sitting less’) were compared with a sedentary lifestyle [12–14]. However, if such sitting less regimens can exert similar beneficial effect on insulin sensitivity and/or involve similar mechanistic pathways is currently unknown.

The mechanisms underlying the beneficial effect of exercise on type 2 diabetes have been well established and involve improvements in skeletal muscle insulin sensitivity and mitochondrial function [15] and extramuscular effects such as lowering of hepatic fat content and concomitant improvements in hepatic insulin sensitivity. Using a sophisticated multi-omics approach, it was recently shown that acute exercise leads to a wide range of molecular adaptations of biological processes such as energy metabolism, oxidative stress, inflammation, tissue repair and growth factor response in plasma and peripheral blood mononuclear cell (PBMCs) but responses in skeletal muscle have not yet been investigated [16] and it remains unclear to what extent such adaptations can also be achieved by low-intensity activities such as standing and walking. Here, we investigated whether a sitting less regimen (i.e. exchanging sedentary time with low-level physical activities such as walking and standing) would be a promising and realistic tool as an alternative to exercise in the battle against insulin resistance. We compared metabolomics and physiological responses to 4 days of acute exercise, sitting less and/or sedentary behaviour in insulin-resistant, obese postmenopausal women.

## Methods

### Participants

Sample size was based on the primary endpoint, which was the glucose rate of disappearance (*R*_d_) measured during a two-step hyperinsulinaemic–euglycaemic clamp. Using this method, we previously found a mean ± SD *R*_d_ of 33.82 ± 7.68 μmol kg^−1^ min^−1^ during placebo conditions and an SD *R*_d_ of the difference between two measurements in the same person was 2.48 μmol kg^−1^ min^−1^. An estimated effect size of 8% between sit and sit less, based on results from our previous SITLESS study [12–14], resulted in 12 participants needed to detect a difference of 2.70 μmol kg^−1^ min^−1^ (8%) between the sit and sit less regimens with a power of 80% and a Bonferonni corrected α < 0.05 using a paired *t* test. Recruitment and data collection took place between December 2017 and March 2020 in the vicinity of Maastricht. In total 27 female volunteers were screened, of whom 14 were found eligible and were included in the study. Of these, 12 participants completed the study (one participant dropped out due to personal reasons and one dropped out because of COVID-19 regulations). The screening included an assessment of blood biochemistry, electrocardiography, anthropometry measurements and a questionnaire including the Baecke physical activity questionnaire [17]. Inclusion criteria were as follows: healthy postmenopausal women (>1 year since last menstrual period); 45–70 years of age; BMI 25–35 kg/m^2^; physically inactive lifestyle (<3 h exercise per week); non-smoking for at least 6 months; alcohol use of ≤2 servings per day; and stable body weight for at least 6 months. We chose to only include women to reduce variability and exclude sex differences.

### Study design

**Fig. 1:**
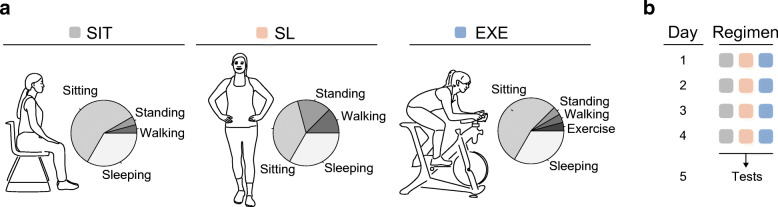
Sitting, sitting less and exercise regimens to assess metabolic health. Regimens for sitting, sitting less and exercise are denoted as SIT, SL and EXE, respectively. (**a**) Visualisation of the three activity regimens. Each participant followed the activity regimens in a random order. A day in the SIT regimen consisted of 1 h walking, 1 h standing, 14 h sitting and 8 h sleeping. A day in the SL regimen consisted of 3 h walking, 4 h standing, 9 h sitting and 8 h sleeping. A day in the EXE regimen consisted of 1 h exercise, 1 h walking, 1 h standing, 13 h sitting and 8 h sleeping. (**b**) Each activity regimen lasted 4 days and measurements were performed on day 5. The activity regimens were separated by a washout period of 9–23 days

A randomised three-arm crossover intervention study was performed, with insulin sensitivity measured by a two-step hyperinsulinaemic–euglycaemic clamp as primary outcome. The design is similar to that used in a previous study [14]. In short, participants (Table 1) randomly underwent all three study arms, each including well-controlled 4 day activity regimens: (1) sitting; (2) sitting less; and (3) exercise (Fig. 1). Prior to the three activity regimens baseline measurements were performed for participant characterisation. During the sit regimen, participants were instructed to sit for 14 h/day, stand for 1 h/day, walk for 1 h/day and to spend 8 h/day sleeping or lying. During the exercise regimen, 1 h/day of sitting was replaced by 1 h/day of moderate-to-vigorous supervised exercise. The rest of the day in the exercise regimen was spent as for the sit regimen. During the sitting less regimen, 5 h/day of sitting was replaced by 3 h of standing and 2 h of walking. Participants were advised to spread the standing and walking hours over the day. The exercise and sitting less regimens differed largely in time spent sitting but were designed to have equal total daily energy expenditure. The sit and exercise regimens differed by only 1 h in sitting time but had substantially different total daily energy expenditure. Any other physical activities besides sitting, standing, walking and the supervised exercise session were limited as much as possible. Directly after the 4 day activity regimens, on day 5, several measurements were performed. A washout period of at least 9 days and maximum 23 days was applied between the activity regimens, and the physical activity of participants was not assessed during this time.

**Table 1:** Participant characteristics

Variable	Mean ± SD^a^
Sex (female/male)	12 / 0
Age (years)	64 ± 5
BMI (kg/m^2^)	29.2 ± 2.9
Fasting glucose (mmol/l)	5.4 ± 0.5
$\dot{V}{\mathrm{O}}_{2\max }$ (ml min^−1^ kg^−1^)	23.8 ± 4.3

^a^Sex data are presented as the number of participants

### Standardisation of dietary intake

Dietary intake was kept similar during all three regimens. Participants were instructed to adhere to their normal dietary habits. The consumption of alcohol and caffeine-rich drinks was not allowed during the five intervention days. During the first activity regimen, participants carefully recorded both time and content of all consumptions in a diary. The researcher returned these dietary records to the participants who were instructed to consume the same diet in the second and third activity regimen. To standardise the last 12 h before this test day, participants were instructed to consume a standardised dinner on the evening of day 4 before 20:00 hours and to refrain from food and drinks afterwards except for water.

### Physical activity assessment

Physical activity and body posture allocation was measured 24 h/day using an activPAL activity monitor (PAL technologies, Glasgow, UK). The activPAL was enclosed in a waterproof pack and attached to the anterior thigh of the participant. Before the start of the actual activity regimens, participants first wore the activPAL monitor for five consecutive days, including three weekdays and two weekend days, to assess habitual physical activity level. The activPAL discriminates between time being inactive (sitting or lying) or being active (standing and walking) and records step number and cadence. In addition, participants recorded their physical activity in a diary and these data were compared with activPAL readouts to formulate tailor-made instructions on how to alter daily activities to those prescribed for the three different activity regimes. This was done to guarantee optimal compliance with each activity regimen. Sleeping time was determined based on diary data. Daily energy expenditure was estimated using the 24 h activPAL data expressed in metabolic equivalents (i.e. metabolic equivalent of task [MET], oxygen uptake of 3.5 ml kg^−1^ min^−1^) and with separate calculations for the exercise session as described earlier [14].

### Participant characteristics

To assess participant characteristics, body mass and body volume were assessed using air-displacement plethysmography using the BodPod device (Cosmed, Rome, Italy) according to the manufacturer’s instructions [18], and body composition (fat mass and fat free mass) was calculated. Waist and hip circumference were measured and the average of three values were reported. Next, in vivo maximal oxygen consumption ($\dot{V}{\mathrm{O}}_{2\max }$, and the corresponding adjusted wattage as Wattmax. The $\dot{V}{\mathrm{O}}_{2\max }$ reflects the participant’s physical fitness and 60% of the Wattmax was applied during the supervised exercise session. All the measurements were performed in the morning after an overnight fast of at least 10 h with a minimum of 3 days before the start of the activity regimens.

### Exercise session

The exercise session consisted of a supervised cycling protocol at 60% of the Wattmax on an ergometer for 45–60 min. The exact cycling time was adjusted per participant based on activPAL recordings, to ensure equal estimated total energy expenditure between the sitting less and exercise regimen. The cycling was divided in repeating blocks of 15 min cycling followed by 2 min rest, until the anticipated total cycling time was reached.

### Intrahepatic lipid quantification by MR spectroscopy

At 07:00 hours on the test day after ending the activity regimen period, proton magnetic resonance spectroscopy (^1^H-MRS) was used to quantify intrahepatic lipid content (IHL). All participants were relocated in a wheelchair from the research facility to the radiology department. Measurements were performed on a 3.0 T whole-body scanner (Achieva Tx; Philips Healthcare, Best, the Netherlands). Spectra were acquired as described previously [20]. Values are given as T2 corrected ratios of CH_2_/(CH_2_+H_2_O) expressed as percentage. One participant failed to undergo this measurement, due to claustrophobia, and another participant was excluded due to a technical error.

### Skeletal muscle biopsies

Following the MRS measurement, a muscle biopsy was taken (08:30 hours) from the vastus lateralis muscle under local anaesthesia (1% lidocaine, without adrenaline [epinephrine]) using the Bergström technique [21]. Muscle tissue was used for mitochondrial respiratory measurements and metabolomics analyses.

### Skeletal muscle mitochondrial respiration

Muscle tissue obtained through the biopsy was immediately placed in ice-cold preservation medium (BIOPS; OROBOROS Instruments, Innsbruck, Austria). After permeabilisation using saponin and several washing steps with an ice-cold mitochondrial respiration buffer (MiR05; OROBOROS Instruments), muscle fibres were transferred into a high-resolution respirometer (Oxygraph; OROBOROS Instruments). Thereafter, ex vivo mitochondrial respiration was determined by measuring $\dot{V}{\mathrm{O}}_2$ (pmol [mg wet weight]^−1^ s^−1^) upon addition of several substrates, as described previously [22]. All measurements were performed in quadruplicate and traces with a cytochrome C response above 15%, indicating disrupted integrity of the inner mitochondrial membrane, were excluded. Respirometry could not be determined in one participant due to difficulties with the muscle biopsy.

### Skeletal muscle metabolomics

Metabolomics was performed as described previously, with minor adjustments [23]. In a 2 ml tube, the following amounts of internal standard dissolved in water were added to each sample of approximately 5 mg of freeze-dried muscle tissue: 5 nmol adenosine ^15^N_5_-monophosphate; 5 nmol adenosine ^15^N_5_-triphosphate; 0.5 nmol d_4_-alanine; 0.5 nmol d_7_-arginine; 0.5 nmol d_3_-aspartic acid; 0.5 nmol d_3_-carnitine; 0.5 nmol d_4_-citric acid; 0.5 nmol ^13^C_1_-citrulline; 1 nmol ^13^C_6_-fructose 1,6-diphosphate; 5 nmol guanosine ^15^N_5_-monophosphate; 5 nmol guanosine ^15^N_5_-triphosphate; 10 nmol ^13^C_6_-glucose; 1 nmol ^13^C_6_-glucose 6-phosphate; 0.5 nmol d_3_-glutamic acid; 0.5 nmol d_5_-glutamine; 1 nmol d_5_-glutathione; 0.5 nmol ^13^C_6_-isoleucine; 1 nmol d_3_-lactic acid; 0.5 nmol d_3_-leucine; 0.5 nmol d_4_-lysine; 0.5 nmol d_3_-methionine; 0.5 nmol d_6_-ornithine; 0.5 nmol d_5_-phenylalanine; 0.5 nmol d_7_-proline; 0.5 nmol ^13^C_3_-pyruvate; 0.5 nmol d_3_-serine; 0.5 nmol d_6_-succinic acid; 0.5 nmol d_5_-tryptophan; 0.5 nmol d_4_-tyrosine; and 0.5 nmol d_8_-valine. After adding the internal standard mix, a stainless-steel bead (5 mm) and polar phase solvents (for a total of 500 μl water and 500 μl methanol) were added and samples were homogenised using a TissueLyser II (Qiagen, Germany) for 5 min at a frequency of 30/s. Chloroform was added (to a total of 1 ml) to each sample before thorough mixing. Samples were then centrifuged for 10 min at 20,000 *g*. The top layer, containing the polar phase, was transferred to a new 1.5 ml tube and dried using a vacuum concentrator at 60°C. Dried samples were reconstituted in 100 μl 3:2 (vol./vol.) methanol–water. Metabolites were analysed using a Waters (the Netherlands) Acquity ultra-high-performance LC system coupled to a Bruker (USA) Impact II Ultra-High Resolution Qq-Time-Of-Flight mass spectrometer. Samples were kept at 12°C during analysis and 5 μl of each sample was injected. Chromatographic separation was achieved using a Merck Millipore (USA) SeQuant ZIC-cHILIC column (PEEK 100 × 2.1 mm, 3 μm particle size). Column temperature was held at 30 °C*.* Mobile phase consisted of (A) 1:9 (vol./vol.) acetonitrile–water and (B) 9:1 (vol./vol.) acetonitrile–water, both containing 5 mmol/l ammonium acetate. Using a flow rate of 0.25 ml/min, the LC gradient consisted of 100% B for 0–2 min, reaching 0% B at 28 min, 0% B for 28–30 min, reaching 100% B at 31 min, and 100% B for 31–32 min. Column re-equilibration was achieved at a flow rate of 0.4 ml/min at 100% B for 32–35 min. MS data were acquired using negative and positive ionisation in full scan mode over the range of m/z 50–1200. Data were analysed using Bruker TASQ software version 2.1.22.3. All reported metabolite intensities were normalised to dry tissue weight, as well as to internal standards with comparable retention times and response in the MS. Metabolite identification was based on a combination of accurate mass, (relative) retention times and fragmentation spectra, compared with the analysis of a library of standards.

### Plasma triacylglycerols

Triacylglycerols (Sigma, Zwijndrecht, the Netherlands) were analysed using a Pentra 400 in EDTA plasma from fasting blood samples taken in the morning between 06:30 hours and 07:00 hours.

### Hyperinsulinaemic–euglycaemic clamp

After the ^1^H-MRS, muscle biopsy and BP measurement, a two-step hyperinsulinaemic–euglycaemic clamp was performed to determine hepatic and peripheral insulin sensitivity [24]. During the ^1^H-MRS measurement (at 07:00 hours) a primed, continuous d-6.6-[^2^H_2_]glucose (d_2_-glucose) tracer infusion was started (0.04 mg kg^−1^ min^−1^). Upon 3 h of d_2_-glucose pre-infusion, a 3 h primed low-dose continuous insulin infusion was started (10 mU m^−2^ min^−1^) to assess hepatic insulin sensitivity. Subsequently, a 2.5 h primed high-dose continuous insulin infusion (40 mU m^−2^ min^−1^) was started, to measure peripheral insulin sensitivity. Blood was frequently sampled from arterialised blood to monitor glucose levels required to maintain euglycaemia (~5.0–5.5 mmol/l). During the last 30 min of the baseline, low and high insulin phases, steady state was reached and blood samples were collected for determination of glucose tracer kinetics. During these phases whole-body substrate utilisation was also measured using indirect calorimetry (Omnical; Maastricht Instruments, Maastricht, the Netherlands) [25]. Due to technical reasons, one participant was excluded from the low insulin phase clamp analysis and two participants were excluded from the high insulin phase clamp analysis.

### Calculations for physiological measurements

During the clamp, energy expenditure, carbohydrate oxidation and fat oxidation rates were calculated using equations based on the measured oxygen and carbon dioxide concentrations, with the assumption that protein oxidation was negligible [26, 27]. The respiratory exchange ratio (RER) was calculated by the carbon dioxide/oxygen ratio. Steele’s single pool non-steady state equations were used to correct for small differences in glucose concentrations, to calculate glucose rate of appearance (*R*_a_) and rate of disappearance (*R*_d_) [28]. Volume of distribution was assumed to be 0.160 l/kg for glucose. Insulin-stimulated glucose *R*_d_ was calculated during the low and the high insulin infusion. Endogenous glucose production (EGP) was calculated as *R*_a_ minus exogenous glucose infusion rate. The percentage of insulin-suppressed EGP was calculated as the percentage of insulin-suppressed EGP over the basal EGP. Non-oxidative glucose disposal (NOGD) was calculated as *R*_d_ minus carbohydrate oxidation.

### Statistical analyses

Participant characteristics are reported as mean ± SD; other outcome measures are reported as mean ± SE. Data are presented for *n* = 12, unless otherwise indicated. All data were evaluated for normal distribution. Differences between interventions were analysed with a repeated measures ANOVA for parametric data or with a Friedman test for non-parametric data. Post hoc Bonferroni and Dunn’s correction, respectively, was applied to correct for multiple testing and to adjust the *p* values. Statistical significance was set at *p* < 0.05 on these adjusted *p* values. Statistical analyses were performed using IBM SPSS version 23.0 for MacOS. For metabolomic data, analyses were performed with R [29] version 3.5.1 and Bioconductor [30] version 3.7. Partial least-squares discriminant analysis (PLS-DA) and metabolite variable importance to projections (VIP) scores were calculated using the R package MixOmics [31] version 6.6.2. Significance was assessed using an empirical Bayes moderated *t* test on log_2_ transformed data within limma’s linear model framework, taking participants and their regimen into account [32, 33]. Unless implemented through an aforementioned R package or base R graphics, visualisation of data was performed using ggplot2 [34].

### Ethical approval

The study was conducted in accordance with the principles of the declaration of Helsinki and approved by the Ethics Committee of the Maastricht University Medical Center. The study was registered at https://clinicaltrials.gov (NCT03912922). All participants provided written informed consent before screening.

## Results

### Sitting less and exercise regimens to assess metabolic health

Twelve healthy overweight and obese women (age 64 ± 5 years, BMI 29.2 ± 2.9 kg/m^2^) participated and completed the study (Table 1, Fig. 1). Participants were non-smokers, had no active diseases, and had a sedentary lifestyle according to the Baecke questionnaire (6.55 ± 0.95) (electronic supplementary material [ESM] Table 1). Medication use by the participants was not expected to interfere with the main outcomes of this study (ESM Table 2). Habitual daily physical activity level showed a mean habitual sedentary time (including sleeping time) of 17.6 ± 1.2 h, standing time 4.3 ± 1.2 h and walking time 2.1 ± 0.6 h, with a mean of 10,141 ± 3250 steps taken (ESM Table 1).

The three activity regimens were successfully implemented, as the time spent sitting, standing, walking and exercising were in accordance with the study design (ESM Table 3). The time spent standing (4.0 ± 0.1 h) and walking (3.0 ± 0.1 h) were significantly higher in the sitting less regimen compared with the sitting (*p* < 0.01) and exercise regimens (*p* < 0.01) (ESM Table 3). The times spent standing (sitting regimen 1.3 ± 0.1 h, exercise regimen 1.2 ± 0.1 h, *p* = 0.99) and walking (sitting regimen 1.0 ± 0.0 h, exercise regimen 1.0 ± 0.0 h) were not significantly different between the sitting and exercise regimens (ESM Table 3). During the exercise regimen, a mean of 1.0 ± 0.1 h of sitting per day was substituted by cycling during a supervised exercise session at a mean intensity of 82 ± 5 W, corresponding to a mean of 4.60 ± 0.15 METs. The number of steps per day was significantly higher in the sitting less regimen (16,875 ± 463 steps/day) compared with the sitting (4878 ± 240 steps/day, *p* < 0.01) and exercise regimen (5082 ± 165 steps/day, *p* < 0.01) (ESM Table 3). Furthermore, the walking cadence was significantly higher in the sitting less regimen (94 ± 2 steps/min) compared with the sitting (85 ± 3 steps/min, *p* < 0.01) and exercise (86 ± 2 steps/min, *p* < 0.01) regimens (ESM Table 3). The estimated energy expenditure was lower in the sitting regimen (32.0 ± 0.1 MET/day) compared with the exercise (36.6 ± 0.2 MET/day, *p* < 0.0001) and sitting less regimens (37.0 ± 0.2 MET/day, *p* < 0.01) (ESM Table 3). The amount of time spent sleeping did not differ between the three regimens, with a mean of 8.0 ± 0.1 h/day (*p* = 0.28) (ESM Table 3). The self-reported dietary intake did not differ between the three activity regimens.

### Sitting less and exercise improve insulin sensitivity compared with sitting

**Fig. 2:**
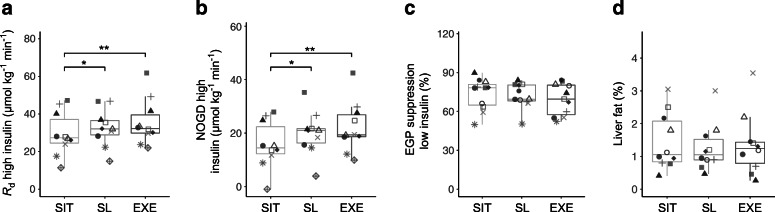
The sitting less and exercise regimens improve insulin sensitivity compared with the sitting regimen. Regimens for sitting, sitting less and exercise are denoted as SIT, SL and EXE, respectively. Data are shown by boxplots in which each participant is represented by a particular symbol throughout the fig. A hyperinsulinaemic–euglycaemic two-step clamp was performed to assess insulin sensitivity. (**a**) Whole-body insulin-stimulated glucose disposal during high-dose insulin infusion expressed as *R*_d_ (*n* = 10). (**b**) NOGD during high-dose insulin infusion (*n* = 10). (**c**) Suppression of hepatic EGP during low-dose insulin infusion (*n* = 11). (**d**) IHL measured by ^1^H-MRS (*n* = 10). *p < 0.05 and **p < 0.01. Boxplots: box includes the IQR corresponding to 25th percentile (Q1, bottom of box), 50th percentile (Q2, bold line within box, i.e. the median) and 75th percentile (Q3, top of box) of the data. The bottom whisker is set at Q1 – 1.5 × IQR and the top whisker at Q3 + 1.5 × IQR

We examined the effects of the sitting, sitting less and exercise regimens on whole-body and hepatic insulin sensitivity using a two-step hyperinsulinaemic–euglycaemic clamp. Fasting plasma glucose levels measured before the start of the clamp did not differ between the three regimens (sitting 5.4 ± 0.1, sitting less 5.4 ± 0.1, exercise 5.5 ± 0.1 mmol/l, *p* = 0.29). The glucose *R*_d_ during high rate of insulin infusion, reflecting peripheral (mainly muscle) insulin sensitivity, was significantly higher in the sitting less (33.1 ± 3.2 μmol kg^−1^ min^−1^, *p* = 0.03) and exercise (35.2 ± 3.8 μmol kg^−1^ min^−1^, *p* < 0.01) regimens compared with the sitting regimen (29.4 ± 3.7 μmol kg^−1^ min^−1^) (Fig. 2a and ESM Table 4). Therefore, this demonstrates a significant improvement in peripheral insulin sensitivity in the sitting less (~13%) and the exercise regimen (~20%), compared with the sitting regimen. The higher peripheral insulin sensitivity was mainly due to a significantly higher NOGD in the sitting less (19.9 ± 2.6 μmol kg^−1^ min^−1^, *p* = 0.04) and exercise regimens (22.3 ± 3.0 μmol kg^−1^ min^−1^, *p* < 0.01) compared with the sitting regimen (15.7 ± 2.8 μmol kg^−1^ min^−1^) (Fig. 2b and ESM Table 4). Insulin-stimulated carbohydrate oxidation remained similar in the three activity regimens (ESM Table 4). EGP suppression during the low insulin phase, reflecting hepatic insulin sensitivity, was similar after all three activity regimens (Fig. 2c and ESM Table 4). IHL determined by MRS was unaffected by the activity regimen (*p* = 0.68) (Fig. 2d), despite a (non-significantly) lower fasting plasma triacylglycerol level in the exercise and sitting less regimens compared with the sitting regimen (exercise 0.81 ± 0.12, sitting less 0.83 ± 0.09, sitting 1.01 ± 0.15 mmol/l, exercise vs sitting *p* = 0.04, sitting less vs sitting *p* = 0.13, exercise vs sitting less *p* = 0.99). The baseline RER measured during the hyperinsulinaemic–euglycaemic clamp was non-significantly lower in the sitting less regimen compared with the sitting regimen (sitting less 0.76 ± 0.01, sitting 0.78 ± 0.01, *p* = 0.10) (ESM Table 4). However, baseline fat oxidation and carbohydrate oxidation were not different between activity regimens (*p* = 0.19 and *p* = 0.24 respectively, ESM Table 4). In addition, fat oxidation, carbohydrate oxidation and RER during insulin stimulation were not affected by the activity regimes (ESM Table 4).

### Sitting less induces metabolic changes at the molecular level similar to the changes induced by exercise

The beneficial effects of exercise on metabolic health involve major molecular adaptations in skeletal muscle. We therefore investigated whether the sitting less regimen would lead to similar molecular events in muscle compared with exercise. First, mitochondrial respiratory capacity was measured in skeletal muscle biopsies. ADP-stimulated (state 3) respiration upon complex I-linked substrates (MG3) and upon lipid-derived substrates (MO3) were similar between the three activity regimens (ESM Table 5). Similar results were observed upon parallel electron input to both complex I and II by sequentially adding the complex II-linked substrate succinate (ESM Table 5). Maximal trifluoromethoxy carbonylcyanide phenylhydrazone (FCCP)-induced uncoupled respiration, reflecting the maximal capacity of the electron transport chain, was also unchanged (ESM Table 5). Finally, mitochondrial respiration upon the treatment with ATP synthase inhibitor oligomycin, reflecting leak respiration, was similar between the three activity regimens (ESM Table 5).

**Fig. 3:**
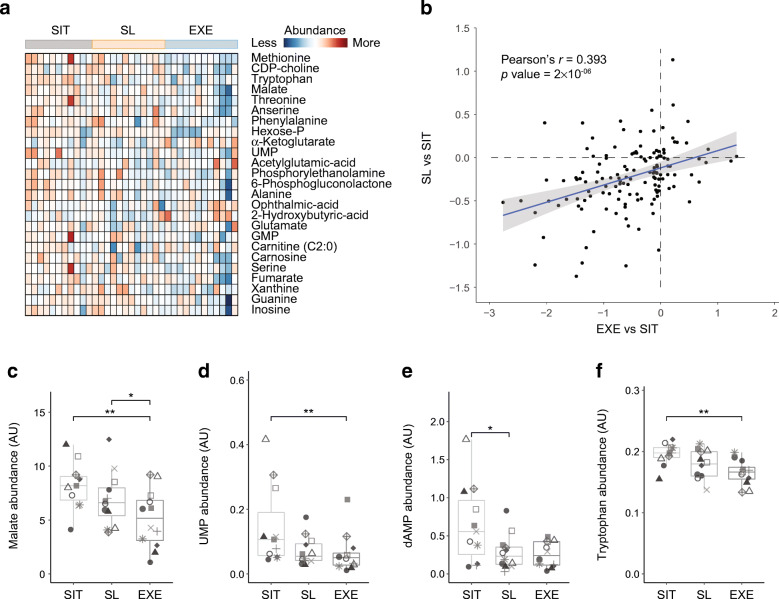
Responses to sitting less are similar to those produced by exercise at the molecular metabolic level in skeletal muscle. Regimens for sitting, sitting less and exercise are denoted as SIT, SL and EXE, respectively. Metabolomics analyses were performed in skeletal muscle biopsy samples of participants (*n* = 11). Heatmap and box plots show stepwise metabolite abundance changes going from SIT to SL to EXE. (**a**) Heatmap of the top 25 metabolites, ranked by their VIP scores from the PLS-DA (ESM Fig. 1a). Relative abundance levels in each participant per regimen is scaled from low abundance (blue) to high abundance (red). (**b**) Comparison of the significance of differences that EXE induces (compared with sitting), relative to differences induced by SL (compared with sitting). Units on the axes are *p* values on a −log_10_ scale. Directionality of induced changes are represented as either negative values (decreased) or positive values (increased). Pearson’s *r* = 0.393, *p* = 2 × 10^−6^. (**c**–**f**) The metabolomic shift induced by SL and EXE is illustrated for malate (**c**), uridylic acid (**d**), deoxyadenosine monophosphate (**e**) and tryptophan (**f**). Data are shown by boxplots in which each participant is represented by a particular symbol throughout the figure. Significance was determined using an empirical Bayes moderated *t* test in a linear model framework. *p < 0.05 and **p < 0.01. AU, arbitrary units; CDP-choline, cytidine 5′-diphosphocholine; dAMP, deoxyadenosine monophosphate; Hexose-P, hexose phosphate; UMP, uridylic acid. Boxplots: box includes the IQR corresponding to 25th percentile (Q1, bottom of box), 50th percentile (Q2, bold line within box, i.e. the median) and 75th percentile (Q3, top of box) of the data. The bottom whisker is set at Q1 – 1.5 × IQR and the top whisker at Q3 + 1.5 × IQR

Next, we investigated molecular metabolic changes by using semi-targeted metabolomics with ultra-high-performance LC coupled to high-resolution MS (UPLC-HRMS) on the muscle biopsies of the participants. With this method we were able to detect a total of 138 metabolites. As a first assessment of the global differences between intervention regimens, we performed PLS-DA. We observed greatest separation of the exercise regimen from the other two regimens within the first component, with separation of the sitting less regimen compared with the sitting regimen becoming more apparent within the second component (ESM Fig. 1a). To further explore the differences between these three regimens, we next ranked metabolites for their contribution to this separation, using their VIP scores derived from the PLS-DA. Selecting the top 25 metabolites and visualising their abundances per individual suggested that sitting less represented an intermediary state between sitting and exercise (Fig. 3a).

To fully evaluate how closely the sitting less regimen resembled exercise relative to sitting, we calculated the fold changes and significance levels of each metabolite in a linear framework model, taking each regimen and individual into account. Comparing fold change with significance level in a Volcano plot indeed demonstrated that exercise induced a greater amount of molecular metabolic changes (ESM Fig. 1b–d). We next assessed whether the changes induced by exercise were the same as those induced by the sitting less regimen, when compared with just sitting. We found a significant positive correlation between the exercise and sitting less regimens (Pearson’s *r* = 0.393 *p* = 2 × 10^−6^) (Fig. 3b). Identifying metabolites that were most exemplary of this correlation (ESM Fig. 1e) showed a stepwise association of abundance levels in metabolites, changing in abundance from sitting, to sitting less, to exercise; as exemplified by malate, uridylic acid, deoxyadenosine monophosphate, tryptophan, methionine and threonine, among others (Fig. 3c–f and ESM Fig. 1f,g). Taken together, these findings suggest that at the molecular metabolic level, although less pronounced, sitting less induced similar types of metabolic changes to those induced by exercise.

## Discussion

Sedentary behaviour is considered a major cause of metabolic disease and regular exercise is the superior intervention for preventing this. However, long-term adherence to exercise programmes is often low. Recently, it has been suggested that reducing sedentary behaviour per se can improve markers of glucose homeostasis [7–14], although the underlying mechanisms and a direct comparison with exercise are lacking. Here, we show that replacing sitting time by standing and walking (i.e. sitting less) for 4 days had similar beneficial effects as daily exercise on insulin sensitivity, as measured by the gold standard hyperinsulinaemic–euglycaemic clamp technique. Furthermore, analysis of muscle biopsies showed that sitting less shifted the molecular metabolic profile towards that of moderate-to-vigorous exercise, suggesting partly overlapping mechanisms. We conclude that replacing sitting time with standing and walking is an attractive and highly feasible alternative to moderate-to-vigorous exercise to improve metabolic health.

Several studies have shown that reducing sedentary behaviour improves glucose homeostasis measured by an OGTT [12–14] or by attenuated postprandial plasma glucose and insulin responses during meal tests [7–11]. While OGTTs and meal tests give valuable information about glucose homeostasis, the gold standard for assessing human insulin sensitivity, the hyperinsulinaemic–euglycaemic clamp developed by DeFronzo et al. [35], has only been used once to investigate the effects of sedentary behaviour on peripheral insulin sensitivity [36]. In that study, healthy young participants reduced their walking activity from a habitual ~10,500 steps per day to ~1400 steps per day for 2 weeks, resulting in a decrease in whole-body insulin sensitivity of ~17% [36]. However, the effects of acute exercise or a low level of physical activity were not investigated. Here, we report a significant improvement in peripheral insulin sensitivity in the sitting less (~13%) and the exercise regimen (~20%), compared with the sitting regimen. Peripheral insulin sensitivity predominantly resides within skeletal muscle and we showed specifically that the improved peripheral insulin sensitivity originated from increased insulin-stimulated glycogen storage (i.e. NOGD) rather than enhanced glucose oxidation. The molecular metabolic changes assessed by UPLC-HRMS-based metabolomics revealed that sitting less shifted the molecular metabolic profile of skeletal muscle towards the profile associated with moderate-to-vigorous exercise. The similarity in metabolic signatures emerging from the sitting less and exercise regimen suggests that the improved skeletal muscle insulin sensitivity observed in both regimens originates from similar metabolic changes. Therefore, reducing sitting time may form a potential effective strategy in the prevention or treatment of type 2 diabetes.

It has previously been shown by us [37] and others [38] that skeletal muscle insulin resistance is associated with a reduction in mitochondrial function. Although it is generally accepted that exercise training exerts positive effects on muscle mitochondrial function and biogenesis [15, 39], and that this partly underlies long-term training-induced improvements in peripheral insulin sensitivity [38, 40], we did not observe an effect of acute exercise and/or the sitting less regimen on muscle mitochondrial function, as measured by respiratory capacity. The duration of the current study (4 days) might have been too short to detect such effects [41]. Regardless, metabolomics analyses in skeletal muscle biopsies did reveal similar molecular metabolic changes with the sitting less and exercise regimen, compared with the sitting regimen. We observed that many changes occurred in a dose-like fashion in the regimens, with the exercise regimen providing the most profound changes compared with the sitting regimen, and the sitting less regimen providing intermediate responses.

The dose-like effect of the sitting less and exercise regimens, compared with the sitting regimen, was marked by a clear reduction in the levels of certain amino acids (e.g. tryptophan, methionine, alanine, threonine, phenylalanine). In line with this, it was recently shown using a multi-omics approach in plasma and PBMCs that a reduction in amino acids is triggered as an early adaptation to acute exercise [16]. These findings are corroborated by another study that observed tryptophan to be markedly reduced in serum during exhaustive aerobic exercise [42] and also a general modulation of amino acids towards lower levels in elite World Tour professional cyclists, with the greatest reduction occurring in cyclists with the best exercise ranking [43]. Additionally, amino acid concentrations in skeletal muscle have been reported to be lower after a long duration of moderate-intensity exercise, although they may be higher after short-duration high-intensity exercise [44]. Our findings in skeletal muscle support the idea that choreography of molecular metabolic adaptations to moderate-intensity exercise involves a reduction in many amino acids and demonstrates that sitting less produces the same pattern towards triggering these effects. Taken together, these results indicate that, as with exercise, sitting less profoundly modulates the skeletal muscle metabolome. Longer-term studies are needed to investigate whether these changes in metabolic profile in muscle translate into changes in functional metabolic adaptations (e.g. improved mitochondrial respiration) and the functional consequences thereof (e.g. improved lipid oxidation and metabolic flexibility), such as have been reported with long-term insulin-sensitising exercise training programmes. Furthermore, functional studies would be required to answer the fascinating question that remains, of whether the depletion of amino acids observed in the exercise regimen and to a lesser extent the sitting less regimen, is a cause or a consequence of the benefits of these regimens.

The beneficial health effects of exercise extend beyond skeletal muscle and include reductions in cardiovascular risk markers such as circulating triacylglycerols and hepatic fat content. Here, we confirm the previously reported [12–14] reduction in fasting plasma triacylglycerol levels resulting from the exercise and, to a lesser extent, sitting less regimen compared with the sitting regimen. These effects are unlikely to originate from alterations in hepatic fat content, as IHL was not affected by the three activity regimens and the lack of change in IHL was reflected by an absence of change in hepatic insulin sensitivity. This finding contrasts with the reduction in IHL found upon prolonged resistance and endurance exercise training [45]. Again, the lack of an effect of any of the exercise regimens on hepatic fat content may originate from the short duration of this intervention (4 days). Alternatively, lower triacylglycerol levels upon exercise may be caused by enhanced clearance of triacylglycerols in the muscle via stimulated lipoprotein lipase activity [46, 47]. Rodent studies support this notion by showing that muscle lipoprotein lipase activity is low upon inactivity but can be increased upon low-intensity muscle contractions, in this case walking slowly [48]. Future studies are needed to test this hypothesis.

One additional point to consider in the interpretation of our results is that the women in our study, while overweight and obese, had a relatively high number of steps (~10,000) per day at baseline. Therefore, both the sitting and the sitting less regimen could be considered as deviations from baseline, representing a period of, respectively, lower and higher activity compared with the participants’ habitual step count. As such, the sitting regimen may have reduced insulin sensitivity compared with their normal levels. Therefore our work could also be interpreted as demonstrating that if active individuals must reduce their habitual activity for whatever reason (e.g. travel, work, etc.), then the harmful effects of this can be reduced by sitting less during that period to mimic their habitual behaviour, or by undertaking a bout of continuous exercise.

### Strengths and limitations

Several strengths and limitations should be considered alongside our findings. First, our work entailed an experimental trial to assess an intervention of sitting less and exercising, relative to a situation of sitting more, in postmenopausal women. The generalisability of these results to other populations would therefore require additional studies. Second, the time between the last exercise or sitting less session and the muscle biopsy collection was <24 h. Therefore, the metabolomic profiles may in part reflect acute responses towards exercise and sitting less, next to early chronic adaptations, which limits the use of these data for making conclusions on the longer-term molecular adaptations to these regimens; however, the similarity in these acute/early chronic adaptations supports the overall conclusion that these regimens elicit similar molecular responses in skeletal muscle. Third, in this experimental trial we performed many measurements, increasing the risk of finding statistical differences. Nonetheless, the primary outcome of the clinical trial, insulin sensitivity, was significant on its own and was intended to be assessed notwithstanding the number of other measures. Additionally, the primary molecular metabolic readout, metabolomics, was significant on its own when comparing the global pattern of whether sitting less and exercise elicited similar responses. Finally, this study was performed on a low number of individuals and future studies may benefit statistically by including a larger number of individuals. A strength of our study is that we confirm the benefits of sitting less and exercise relative to sitting, which we previously reported in lean individuals [13], obese individuals [12] and people with type 2 diabetes [14], showing that irrespective of the number of participants and their particular demographics, sitting less may have beneficial health effects. Here, we added a molecular and mechanistic layer to these previous studies, further providing evidence that sitting less may be an attractive alternative to moderate-to-vigorous exercise for improving metabolic health.

In conclusion, we show here that replacing sedentary time by light physical activities, such as standing and walking, is effective in improving peripheral insulin sensitivity. Exercise and a sitting less regimen revealed similar physiological effects on insulin sensitivity and similar underlying molecular metabolic changes in skeletal muscle, capturing certain elements of the metabolic profile previously observed in athletes by others [16, 42, 43]. This suggests that the sitting less regimen indeed could shift the metabolome in a direction similar to that seen with exercise. Thus, sustained replacement of sitting time by standing and walking may be an attractive and possibly more feasible alternative to moderate-to-vigorous exercise in improving metabolic health.

## Supplementary Information

ESM (PDF 418 kb)

## Data Availability

The datasets generated during and/or analysed during the current study are available from the corresponding author on reasonable request.

## Abbreviations

EGP: Endogenous glucose production
IHL: Intrahepatic lipid content
MET: Metabolic equivalent of task
MRS: Magnetic resonance spectroscopy
NOGD: Non-oxidative glucose disposal
PBMC: Peripheral blood mononuclear cell
PLS-DA: Partial least-squares discriminant analysis
*R*_a_: Rate of appearance
*R*_d_: Rate of disappearance
RER: Respiratory exchange ratio
UPLC-HRMS: Ultra-high-performance LC coupled to high-resolution MS
VIP: Variable importance to projections
$$ \dot{V}{\mathrm{O}}_2 $$: Oxygen consumption
$\dot{V}{\mathrm{O}}_{2\max }$: Maximal oxygen consumption
