# Supplementary material for: Sitting less elicits metabolic responses similar to exercise and enhances insulin sensitivity in postmenopausal women
Source: Diabetologia. 2021 Sep 12;64(12):2817–28. doi: 10.1007/s00125-021-05558-5 (PMC8435176; doi:10.1007/s00125-021-05558-5)
Supplement: Supplementary file 1 — (PDF 418 kb) [file 125_2021_5558_MOESM1_ESM.pdf]

SUPPLEMENTAL FIGURES AND TABLES

ESM Table 1. Additional participant characteristics

Parameter	Mean ± SD
Body weight (kg)	80.1 ± 9.2
Waist circumference (cm)	97 ± 9
Hip circumference (cm)	110 ± 8
Fat mass (%)	46.1 ± 11.3
Systolic blood pressure	133 ± 16
Diastolic blood pressure	85 ± 9
Physical activity level (Baecke score)	6.55 ± 0.95
Habitual sitting + sleeping time (h)	17.6 ± 1.2
Habitual standing time (h)	4.3 ± 1.2
Habitual walking time (h)	2.1 ± 0.6
Habitual steps/day (n)	10141 ± 3250
Habitual cadence (steps/min)	80 ± 5

ESM Table 2. Medication of participants

Medication	N
Lipid lowering drugs (statins)	2
Blood pressure lowering drugs	1
Glaucoma medication	1

ESM Table 3. Physical activity during activity regimes

Parameter	SIT	SL	EXE	p-value	p-value SIT vs EXE	p-value SIT vs SL	p-value EXE vs SL
Sitting (h/day)	13.6 ± 0.2	9.2 ± 0.2	12.5 ± 0.3	<0.01*	<0.01*	<0.01*	<0.01*
Standing (h/day	1.3 ± 0.1	4.0 ± 0.1	1.2 ± 0.1	<0.01*	0.99	<0.01*	<0.01*
Walking (h/day	1.0 ± 0.0	3.0 ± 0.1	1.0 ± 0.0	<0.01*	0.46	<0.01*	<0.01*
Exercise (h/day)	-	-	1.0 ± 0.1	-	-	-	-
Sleeping (h/day	8.1 ± 0.1	7.9 ± 0.2	8.1 ± 0.1	0.28	0.99	0.83	0.29
Steps/day (n)[1]	4878 ± 240	16875 ± 463	5082 ± 165	<0.01*	0.62	<0.01*	<0.01*
Cadence (steps/min)[1]	85 ± 3	94 ± 2	86 ± 2	<0.01*	0.99	<0.01*	<0.01*
Estimated EE[1] (MET*h/day)	32.0 ± 0.1	37.0 ± 0.2	36.6 ± 0.2	<0.01*	<0.01*	<0.01*	0.38

Abbreviations: SIT, sit regime; EXE, exercise regime; SL, sitting less regime. Data are expressed as mean ± SE. N=12, [1]n=11. *p<0.05.

ESM Table 4. Insulin sensitivity and substrate kinetics

Parameter	SIT	SL	EXE	p-value	p-value SIT vs EXE	p-value SIT vs SL	p-value EXE vs SL
Ra (μmol/kg/min)							
Baseline	7.6 ± 0.4	8.4 ± 0.5	7.3 ± 0.5	0.21	0.99	0.46	0.31
Low insulin[1]	11.9 ± 1.3	11.9 ± 0.7	11.7 ± 1.0	0.73	0.99	0.99	0.99
High insulin[2]	29.8 ± 3.6	32.8 ± 3.2	36.1 ± 3.8	<0.01**	0.01*	0.22	0.79
Rd (μmol/kg/min)							
Baseline	7.3 ± 0.4	7.6 ± 0.6	6.9 ± 0.5	0.56	0.99	0.99	0.92
Low insulin[1]	12.5 ± 1.2	12.3 ± 0.9	11.8 ± 0.9	0.73	0.99	0.99	0.99
High insulin[2]	29.4 ± 3.7	33.1 ± 3.2	35.2 ± 3.8	<0.01**	<0.01**	0.03*	0.99
Delta baseline-low[1]	5.0 ± 1.2	4.7 ± 0.7	4.9 ± 0.8	0.98	0.99	0.99	0.99
Delta baseline-high[2]	20.7 ± 3.7	25.5 ± 2.8	28.2 ± 3.3	<0.01**	<0.01**	0.08#	0.99
EGP (μmol/kg/min)							
Baseline	7.6 ± 0.4	8.4 ± 0.5	7.3 ± 0.5	0.21	0.99	0.46	0.31
Low insulin[1]	2.0 ± 0.2	2.3 ± 0.2	2.3 ± 0.2	0.16	0.60	0.17	0.99
% suppression low[1]	73.6 ± 3.7	72.1 ± 2.9	69.2 ± 3.7	0.98	0.99	0.99	0.99
High insulin[2]	0.4 ± 0.2	0.3 ± 0.1	0.7 ± 0.2	0.71	0.99	0.99	0.99
% suppression high[2]	93.6 ± 2.7	96.4 ± 1.9	90.0 ± 2.9	0.44	0.99	0.99	0.54
NOGD (μmol/kg/min)							
Baseline[1]	2.6 ± 0.6	4.0 ± 0.7	3.0 ± 0.6	0.63	0.99	0.99	0.86
Low insulin[1]	3.9 ± 1.2	4.9 ± 1.0	4.3 ± 0.9	0.63	0.86	0.99	0.99
High insulin[2]	15.7 ± 2.8	19.9 ± 2.6	22.3 ± 3.0	<0.01**	<0.01**	0.04*	0.79
Delta baseline-low[2]	1.0 ± 1.7	1.0 ± 0.7	1.0 ± 0.8	0.60	0.99	0.79	0.99
Delta baseline-high[3]	12.2 ± 2.9	15.0 ± 2.6	18.5 ± 2.6	0.01*	0.01*	0.10#	0.99
Carbohydrate oxidation (μmol/kg/min)							
Baseline[1]	5.0 ± 0.6	4.2 ± 0.7	4.5 ± 1.0	0.24	0.46	0.13	0.91
Low insulin[1]	8.6 ± 0.6	7.5 ± 0.6	7.5 ± 0.8	0.35	0.41	0.86	0.99
High insulin[2]	13.8 ± 1.0	13.7 ± 1.2	13.4 ± 1.1	0.98	0.99	0.99	0.99
Fat oxidation (μmol/kg/min)							
Baseline[1]	3.3 ± 0.2	3.6 ± 0.1	3.7 ± 0.2	0.19	0.33	0.18	0.81
Low insulin[1]	2.3 ± 0.2	2.6 ± 0.2	2.5 ± 0.2	0.26	0.26	0.99	0.99
High insulin[2]	1.1 ± 0.2	1.3 ± 0.2	1.3 ± 0.2	0.51	0.99	0.72	0.99
Respiratory exchange ratio							
Baseline[1]	0.78 ± 0.01	0.76 ± 0.01	0.76 ± 0.01	0.16	0.59	0.10#	0.99
Low insulin[1]	0.84 ± 0.01	0.82 ± 0.01	0.82 ± 0.01	0.20	0.63	0.22	0.99
High insulin[2]	0.92 ± 0.01	0.91 ± 0.01	0.91 ± 0.01	0.63	0.99	0.99	0.99
Delta baseline-low[1]	0.07 ± 0.01	0.06 ± 0.01	0.07 ± 0.01	0.66	0.99	0.99	0.99
Delta baseline-high[2]	0.13 ± 0.01	0.14 ± 0.02	0.14 ± 0.02	0.69	0.99	0.99	0.99

Abbreviations: SIT, sit regime; EXE, exercise regime; SL, sitting less regime; Ra, rate of appearance; Rd, rate of disappearance; EGP, endogenous glucose production; NOGD, non-oxidative glucose disposal Data are expressed as mean ± SE. [1]n=11, [2]n=10, [3]n=9. ** p<0.01, *p<0.05, [#]p<0.10.

ESM Table 5. Ex vivo skeletal muscle mitochondrial respiratory capacity

O2 flux (pmol/mg/s)	SIT	SL	EXE	p-value	p-value SIT vs EXE	p-value SIT vs SL	p-value EXE vs SL
MG3	37.5 ± 2.3	35.8 ± 2.2	38.5 ± 2.7	0.42	0.99	0.69	0.78
MO3	36.1 ± 2.5	36.2 ± 2.9	37.7 ± 2.2	0.66	0.99	0.99	0.99
MOG3	47.1 ± 3.9	44.7 ± 3.3	47.5 ± 2.6	0.54	0.99	0.99	0.84
MGS3	72.1 ± 4.5	67.1 ± 4.5	73.4 ± 3.9	0.25	0.99	0.77	0.52
MOGS3	72.0 ± 6.1	68.0 ± 5.0	71.9 ± 3.2	0.58	0.99	0.99	0.99
FCCP	88.5 ± 8.7	87.8 ± 7.2	93.7 ± 5.8	0.53	0.87	0.99	0.99
Oligomycin	24.5 ± 1.8	23.0 ± 2.1	23.8 ± 1.5	0.53	0.99	0.88	0.99

Abbreviations: SIT, sit regime; EXE, exercise regime; SL, sitting less regime; MG3, malate + glutamate; MO3, malate + octanoyl carnitine + glutamate; MOG3, malate + octanoyl carnitine + glutamate; MGS3, malate + glutamate + succinate; MOGS3, malate + octanoyl carnitine + glutamate + succinate; FCCP, maximal FCCP-induced uncoupled respiration; Oligomycin, oligomycin induced respiration not coupled to ATP synthesis. Data are expressed as mean ± SE in pmol per mg wet weight per second. N=11.

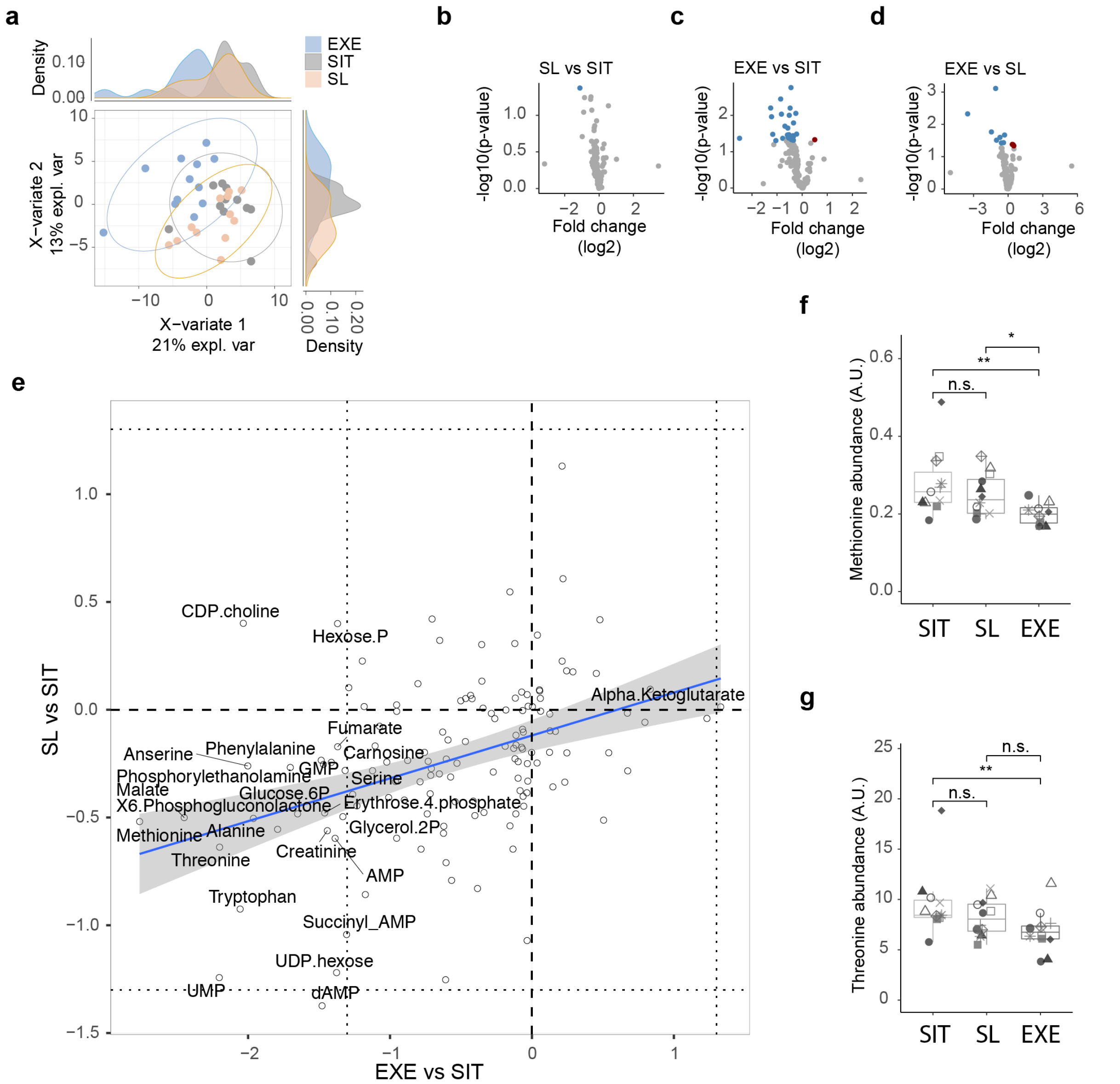

ESM Figure 1: Metabolomic effects of sitting, sitting less, and exercise in skeletal muscle: Regimes for sitting, sitting less, and exercise are denoted as SIT, SL, and EXE, respectively. **a)** Partial least squares discriminant analysis (PLS-DA) on the metabolomes of individuals in the sitting, sitting less, and exercise regimes, n=11, plotting the first component (X-variate 1) and second component (X-variate 2) of the PLS-DA. **b-d**) Volcano plots of fold change (x axis, log2 scale) versus p value (y axis, -log10 scale), for **b**) sitting less vs sit, **c**), exercise vs sit, and **d**) exercise vs sitting less. Reveals exercise to induce the greatest number of metabolic changes. **e**) Highlighted metabolites for plot comparing the significance of differences that exercise induces (compared to sitting), relative to what sitting less induces (compared to sitting). Units on the axes are p values in the –log10 scale. Directionality of induced changes are represented as either negative values (decreased) or positive values (increased). Pearson's r = 0.393, p value = 2e-06 (pertains to main figure 3b). Further visualization of metabolites including **f**) methionine and **g**) threonine illustrates stepwise abundance levels in the sit, sitting less, and exercise regimes. For all panels: *p<0.05, **p<0.01
